# Imaging and CSF analyses effectively distinguish CJD from its mimics

**DOI:** 10.1136/jnnp-2017-316853

**Published:** 2017-11-15

**Authors:** Peter Rudge, Harpreet Hyare, Alison Green, John Collinge, Simon Mead

**Affiliations:** 1 MRC Prion Unit at UCL, UCL Institute of Prion Diseases, London, UK; 2 NHS National Prion Clinic, UCL Hospitals NHS Foundation Trust, London, UK; 3 The National CJD Research and Surveillance Unit, Western General Hospital, London, UK

## Abstract

**Objective:**

To review clinical and investigation findings in patients referred to a specialist prion clinic who were suspected to have sporadic Creutzfeldt-Jakob disease (sCJD) and yet were found to have an alternative final diagnosis.

**Methods:**

Review the clinical findings and investigations in 214 patients enrolled into the UK National Prion Monitoring Cohort Study between October 2008 and November 2015 who had postmortem confirmed sCJD and compare these features with 50 patients referred over the same period who had an alternative final diagnosis (CJD mimics).

**Results:**

Patients with an alternative diagnosis and those with sCJD were of similar age, sex and frequency of dementia but CJD mimics had a longer clinical history. Myoclonus, rigidity and hallucinations were more frequent in patients with sCJD but these features were not helpful in classifying individual patients. Alzheimer’s disease, dementia with Lewy bodies and genetic neurodegenerative disorders were alternative diagnoses in more than half of the CJD mimic cases, and 10% had an immune-mediated encephalopathy; lymphoma, hepatic encephalopathy and progressive multifocal leukoencephalopathy were seen more than once. Diffusion-weighted MRI was the most useful readily available test to classify cases correctly (92% CJD, 2% CJD mimics). The CSF cell count, 14-3-3 protein detection and S100B were of limited value. A positive CSF RT-QuIC test, introduced during the course of the study, was found in 89% of tested CJD cases and 0% CJD mimics.

**Conclusion:**

The combination of diffusion-weighted MRI analysis and CSF RT-QuIC allowed a perfect classification of sCJD versus its mimics in this study.

## Introduction

Although there is no consensus definition for clinical practice, rapidly progressive dementia is commonly considered to comprise a cognitive disorder with progression to advanced stages or death in less than 2 years. Sporadic Creutzfeldt-Jakob disease (sCJD), the most common form of human prion disease, is the prototypic diagnosis of the group, but the differential is wide and includes several treatable disorders. Because of a concern about the potential for iatrogenic spread and zoonoses, specialist prion disease assessment centres are found in many countries and are referred cases suspected to have sCJD and have developed experience in differentiating these from other causes of rapidly progressive dementia.

Among the numerous case reports and series, the two most significant analyses specifically addressing the problem of patients initially diagnosed with sCJD who subsequently had an alternative diagnosis originate from USA and UK.^1 2^ Historically, clinicians have relied on the EEG and abnormal CSF proteins in diagnosing CJD, while being aware that these tests were not highly specific. Since these two papers were published, the imaging features of sCJD have been better characterised and use of diffusion-weighted images has become more universal, enabling the clinician to have greater certainty when diagnosing sCJD.^3 4^ Nevertheless, in spite of the high sensitivity of MRI in the diagnosis of sCJD, the typical findings are frequently not mentioned in the initial radiology report.^5^

Prion diseases are caused by misfolding of a membrane anchored protein, PrP^C^, into abnormal forms, including proteinase resistant forms (PrP^Sc^) and forms which act as a template for PrP^C^ inducing generation of more abnormal PrP.^6^ Capitalising on the templated misfolding mechanism, assays have been developed to detect minute amounts of abnormal PrP that involve cycles of sonication or shaking and incubation with PrP^c^ followed by detection of abnormal PrP by Western blot or thioflavin T binding (PMCA, RT-QuIC).^7 8^ Increasingly the results of the cerebrospinal fluid RT-QuIC assay have been incorporated in epidemiological diagnostic criteria.^9 10^

In this paper, we review the clinical experience of the UK NHS National Prion Clinic in assessment of a large number of patients referred with a provisional diagnosis of sCJD. We compare the clinical and investigative features of those who subsequently were proven to have sCJD at autopsy with those who did not have prion disease and the relative merits of clinical features, imaging, CSF analysis and other tests in classifying patients correctly.

## Methods

Since 2004, patients with a provisional diagnosis of prion disease who are resident in the UK are referred jointly to National CJD Research and Surveillance Unit (Edinburgh, UK) and National Prion Clinic (London, UK). In 2008, the National Prion Monitoring Cohort Study, hereafter called the ‘Cohort Study’, was established; it is an observational longitudinal study of all patients with, or at risk of, developing human prion disease of any type.^11^ All patients are invited to take part in the Cohort Study, which involves review by a member of the National Prion Clinic (consultant neurologist or clinical research fellow) and collection of clinical data including physical examination findings, blood tests, neuropsychology, neurophysiology and MRI at regular intervals depending on the type of prion disease.

**Figure 1:**
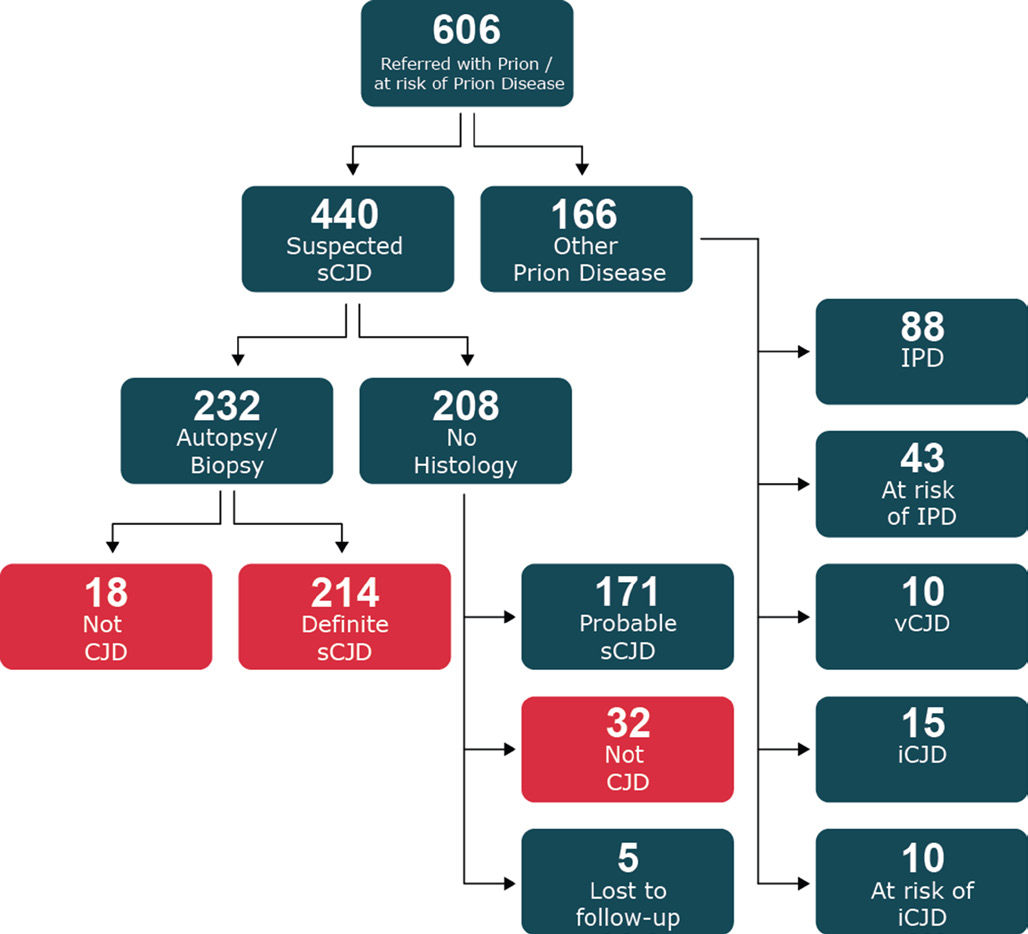
Flow chart of recruitment of all cases suspected of prion disease referred to the National Prion Clinic from 2008 to 2015. Patients included in the current study shown in red. sCJD, sporadic Creutzfeldt-Jakob disease.

In this paper, we review the 606 patients thought on clinical grounds to have prion disease or be at risk of developing prion disease who were referred to the National Prion Clinic and enrolled into the Cohort study between October 2008 and November 2015 (see flow chart in figure 1). Of these, 440 were referred with a clinical diagnosis of suspected sCJD or any other sporadic human prion disease (eg, sporadic fatal insomnia and variably protease sensitive prionopathy). We also included those suspected to have prion disease even though the clinical duration was longer than 2 years. A total of 232 (53%) had an autopsy or cerebral biopsy and 214 were proven to have sCJD and 18 an alternative diagnosis. 208 did not have histological examination of the brain; 171 patients fulfilled the criteria for probable sCJD and 32 had on clinical and investigative grounds an alternative diagnosis. Five additional patients in this group were lost to follow-up.

In this paper, we compared the clinical and investigative features of the 214 definite cases of sCJD with the 50 cases with an alternative diagnosis. None of the 197/214 patients in whom the *PRNP* gene was sequenced had a mutation. All the patients were initially investigated by the referring clinician and virtually all patients had at least one MRI of the brain, usually 1.5T, but sequences varied. The presenting MRI study was reviewed by an experienced neuroradiologist (HH) and an experienced neurologist (PR) and consensus findings were documented. Restricted diffusion in the basal ganglia (caudate, putamen), thalamus and cortex (frontal, parietal, temporal, occipital, insula) was assessed and the presence of atrophy was documented. Finally, any other findings such as white matter lesions and contrast enhancement were noted. CSF was examined in 182 patients and neurodegenerative markers (14-3-3, S100B) sought in 132 patients; RT-QuIC, a technique not in routine clinical use during the early part of the study, was obtained in 75 cases. An EEG report was obtained in 186 patients; in most of these, serial recording was not obtained. Because of the nature of the referrals from other clinicians inevitably there was variation in what other investigations were undertaken. Clinical notes were reviewed and data collected on neuronal and paraneoplastic antibodies, which we would routinely recommend.

Comparisons between groups were made using Student’s t-test, χ² or Fisher’s exact test as appropriate (SPSS, IBM).

## Results

A total of 55 patients referred as suspected cases of sCJD ultimately had an alternative diagnosis to CJD; 26 had a definite alternative diagnosis, 24 had met established consensus clinical criteria for an alternative diagnosis and 5 were lost to follow-up. A total of 214 patients had a definite diagnosis of sCJD at postmortem examination. The demographic features and time to referral of these patients are shown in table 1.

**Table 1:** Demographic features and referral delay in both groups of patients

Diagnosis	Age (years)	Sex ratio M/F	Referral delay (months)mean (median; range)*
sCJD	66.8 (39–85)	98/116	8.8 (3.3; 0.3–22.4)
Not CJD	65.9 (27–88)	22/28	18.8 (34.5; 1.5–108)**

*Note this is delay to referral, not the duration of cognitive decline which was rapid (<2 years) in all.

**P<0.0001.

CJD, Creutzfeldt-Jakob disease; sCJD, sporadic CJD.

The age and sex ratio in the definite patients with CJD and those with an alternative diagnosis were similar. Interestingly, the time before a referral was made to the clinic was much greater in those patients who did not have CJD (8.8 months vs 18.8 months, t-test, P<0.0001).

The most frequent diagnosis was of an alternative neurodegenerative disease, nearly 60% (28 patients) having Alzheimer’s disease (AD) or dementia with Lewy bodies (DLB). The remainder had a variety of other diseases. In 26 patients, the diagnosis was considered certain on the basis of autopsy or definitive laboratory tests and in 24 probable on the basis of clinical picture and investigations.

### Cases with a definite alternative diagnosis

Eight patients were proven to have AD at autopsy, two of whom had in addition minor vascular disease (Table 2). Three were shown at autopsy to have DLB, two of whom had in addition minor vascular disease and one minor AD pathology.

**Table 2:** Final diagnoses in the patients proven not to have CJD

	AD	DLB	Lymphoma PML	Genetic	Antibody mediated	Encephalitis	Hepatic encephalopathy	CVA
Definitive diagnosis	8	3	4	4	5	1	1	0
Clinical and investigation evidence	7	11	0	1	0	3*	1	1

*one case had SSPE

AD, Alzheimer’s disease; CJD, Creutzfeldt-Jakob disease; CVA, cerebrovascular accident; DLB, dementia with Lewy body disease including one case of frontotemporal dementia/motor neuron disease; PML, progressive multifocal leukoencephalopathy; SSPE, subacute sclerosing panencephalitis.

A variety of other definitive diagnoses were made. In six patients in whom autopsies were done two had progressive multifocal leukoencephalopathy (PML), two a cerebral lymphoma, one encephalitis of unknown cause and one hepatic encephalopathy. Nine additional patients who did not have an autopsy were proven to have an alternative diagnosis. Five had an autoimmune encephalopathy due to high titres of Leucine-rich, glioma-inactivated antibodies-1 (LGI1; three cases), N-methyl-D-aspartate antibodies (NMDA; one case) and glycine-1 receptor antibodies (Gly1R-Abs; one case). Four patients had a non-prion genetic cause for their disorder, two carrying hexanucleotide expansions in the *C9orf72* gene, one *CSF1R* mutation (A781V) and one had a combination of beta thalassaemia major and alkaptonuria with a cognitive disorder that improved following withdrawal of nitisinone treatment.^12^

### Cases with a clinical diagnosis

The other 24 patients had good evidence of a clinical diagnosis other than prion disease. Probable DLB (10 patients) was diagnosed by prolonged survival, parkinsonian features, diurnal fluctuating course, predominant visual hallucinations or profound rigidity on administration of dopamine antagonists^13^ and in two a positive DAT scan (Table 2). Probable AD (seven patients) was diagnosed from the prolonged clinical picture dominated by progressive memory impairment and posterior parietal symptoms, absence of other neurological signs, together with MRI (focal hippocampal/posterior parietal atrophy) and CSF markers indicating abnormalities of abeta and tau proteins.^14^ One patient had a frontotemporal dementia with evidence of anterior horn cell disease. Encephalitis (three patients) and stroke were diagnosed from the clinical picture and CSF, blood test results and MRI findings. The patient with hepatic encephalopathy had a spontaneous lienorenal shunt, raised serum ammonia level (106 µmol/L), metal was detected in the lentiform nucleus on T1 MRI, the EEG had abnormal sharpened complexes and there was improvement with shunt closure.

### Features simulating sCJD

The clinical features of the two groups of patients were documented in the Cohort Study systematic neurological history taking and examination (see table 3).

**Table 3:** Summary of symptoms and signs in patients with CJD and non-CJD

Diagnosis	Dementia %	Hallucinations %	Myoclonus %	Rigidity %	Ataxia %
Definite sCJD (214 patients)	99	70	79	72	74
Not CJD (50 patients)	94	49	51	64	72
Fisher’s exact test	P=0.048	P=0.008	P<0.001	P=0.30	P=0.85

CJD, Creutzfeldt-Jakob disease; sCJD, sporadic CJD.

Progressive cognitive decline of less than 2 years duration is an absolute requirement for the diagnosis of probable sCJD and all but two definite sCJD cases in this series fulfilled that criterion. However, in those patients with an alternative diagnosis, although nearly a 94% had cognitive decline, in a substantial proportion, the progression was slower as indicated by the delay in referral (table 1). Most clinicians are aware that patients with sCJD commonly have hallucinations and myoclonus and these were reported in 70%–80% of the present series. These symptoms were significantly less frequent in those with an alternative diagnosis occurring in about half the patients.

Rigidity of the limbs and neck are frequent findings in sCJD but in this series was also a feature in two-thirds of the patients with an alternative diagnosis. Given the frequency of these abnormalities in the patients with an alternative diagnosis, it is not surprising that the referring clinician had CJD in the differential diagnosis; it is in these patients that investigations proved so valuable.

#### Investigations

The results of brain imaging, CSF examination and EEG are shown in table 4.

**Table 4:** Investigation results: (A) MRI, (B) CSF, (C) EEG

A.MRI						
Diagnosis	Normal (%)	Atrophy (%)	Either BG and/or cortex* (%)	BG and cortex* (%)	BG alone* (%)	Cortex alone* (%)
CJD	12/171 (7)	70/178 (40)	158/171 (92)	112/171 (66)	13/171 (8)	33/171 (19)
Not CJD	9/47 (19)	26/47 (55)	2/47 (4)	0	1/47 (2)	1/47 (2)
Fisher’s exact test	P=0.02	P=0.07	P<0.001	P<0.001	P=0.31	P=0.003

B.CSF						
Group	14-3-3 present (%)	S100B elevated (%)	RT-QuIC (%)			
sCJD	112/130 (86)	108/128 (84)	67/75 (89)			
Other diagnosis	13/25 (48)	12/18 (67)	0/25 (0)			
Fisher’s exact test	P<0.001	P=0.094	P<0.001			

C.EEG						
Group	Normal (%)	Slow waves (%)	PSWC (%)			
sCJD	11/186 (6)	154/186† (83)	67/186 (36)			
Other diagnosis	5/40 (13)	25/40 (63)	2/40‡ (5)			
Fisher’s exact test	P<0.17	P=0.009	P<0.001			

*Refers to the consensus opinion of a consultant neuroradiologist and consultant neurologist about the diffusion-weighted images of the BG or cerebral cortex.

†Includes patients who had PSWC.

‡One patient had SSPE type complexes, the other hepatic encephalopathy.

BG, basal ganglia; CJD, Creutzfeldt-Jakob disease; sCJD, sporadic CJD; PSWC, periodic sharp wave complexes; SSPE, subacute sclerosing panencephalitis.

#### Imaging

A total of 171 patients with definite sCJD and 47 patients with other diagnoses had at least one MRI available for review. Seven per cent of the patients with sCJD and 19% of the non-CJD cases had normal imaging (table 4A).

Generalised atrophy was less common in the patients with sCJD (40%) than in the non-prion cases (55%) (P=0.07) but the pattern of atrophy was frequently different. Atrophy was usually generalised in sCJD but it was focal in most neurodegenerative cases including 19 with AD and DLB involving the hippocampus and/or the posterior parietal area, especially in the former. Minor white matter abnormal signal on T2-weighted sequences reflecting vascular disease occurred in about 15% of both groups but extensive white matter abnormality was confined to seven non-prion cases (P<0.001); the diagnoses in these cases were SSPE, lymphoma, PML, *CSF1R* dementia, autoimmune encephalitis and encephalitis of unknown cause.

**Figure 2:**
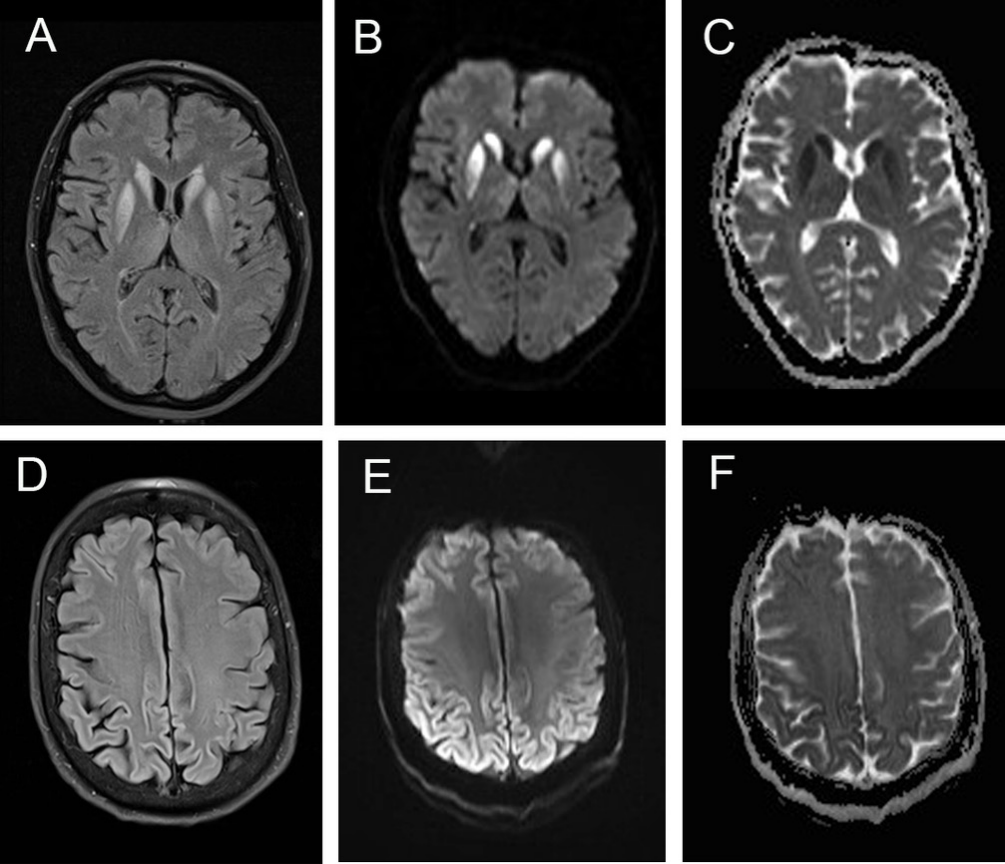
Different MRI sequences in patients with definite sCJD showing the value of DWI confirmed on ADC measurement compared to FLAIR. Images A–C illustrate typical basal ganglia abnormalities on FLAIR (A), DWI (B) and ADC (C). Images E and F show typical features of ‘cortical ribboning’ in the frontal and parietal cortex on FLAIR (D), DWI (E) and ADC (F) sequences. ADC, apparent diffusion coefficient; DWI, diffusion-weighted images; sCJD, sporadic Creutzfeldt-Jakob disease.

The most striking difference between the scans of the two groups was on diffusion weighted sequences (table 4A). There was diffusion restriction in 92% cases of sCJD; 112 (66%) had restriction of diffusion in the basal ganglia and cortex, 13 (8%) had restriction in the basal ganglia alone (figure 2A–C) and 33 (19%) had restriction confined to two or more areas of the cortex (figure 2D–F). Just over half the patients with sCJD had restricted diffusion in the thalamus in one of which the pulvinar signal was prominent.

**Figure 3:**
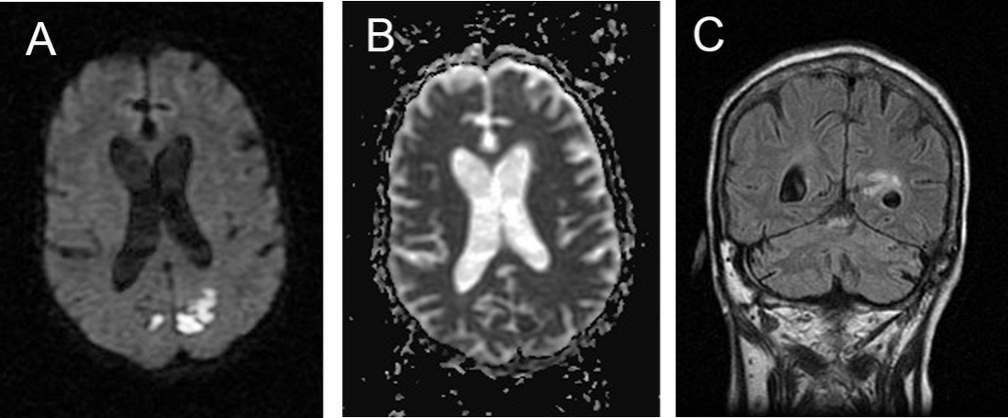
MRI in patient with cerebral lymphoma. Note patchy restricted diffusion in the posterior cortex (A) with partial confirmation on ADC (B) together with abnormal signal in the periventricular area and white matter on FLAIR (C). These areas corresponded to leptomeningeal nodular enhancing soft tissue. ADC, apparent diffusion coefficient.

**Figure 4:**
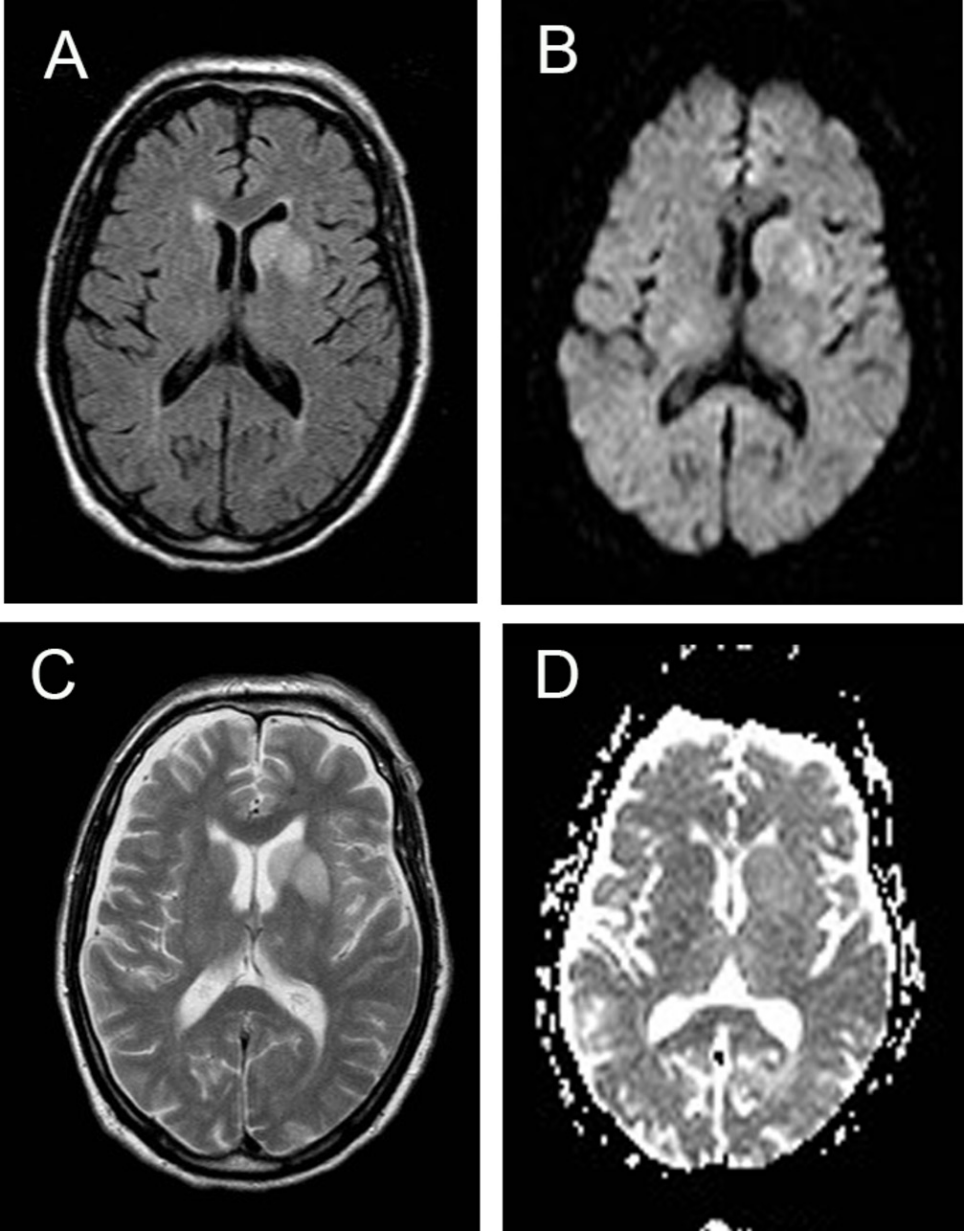
MRI in patient with autoimmune encephalopathy due to LGI1 antibodies. Note the high signal return from the swollen left basal ganglia on T2 (A), FLAIR (B) and DWI (C) sequences but restriction is not confirmed on ADC mapping (D). ADC, apparent diffusion coefficient; DWI, diffusion-weighted images; LGI1, Leucine-rich, glioma-inactivated antibodies-1.

**Figure 5:**
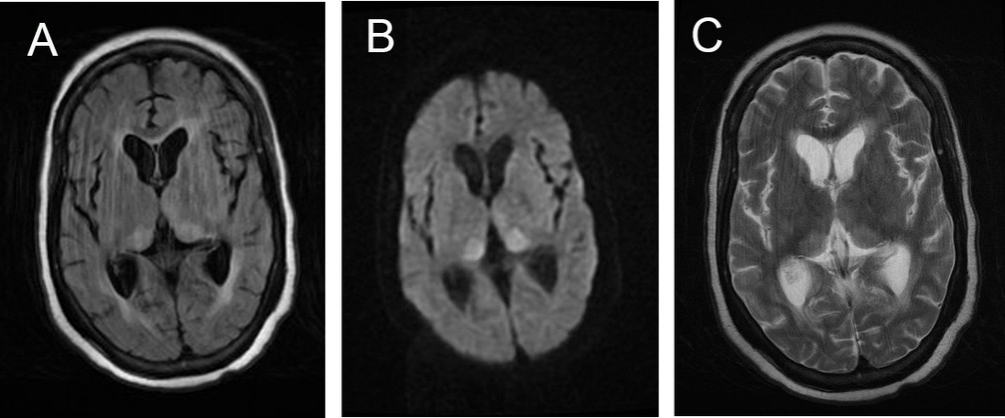
MRI in a patient with autoimmune encephalopathy due to NMDA antibodies is similar to the scans seen in vCJD. Note pulvinar sign on FLAIR (A) and DWI (B). The ADC scan did not confirm restriction (C). There is minor periventricular high signal in the FLAIR sequence (A). ADC, apparent diffusion coefficient; DWI, diffusion-weighted images; NMDA, N-methyl-D-aspartate antibodies.

In contrast, diffusion abnormalities, confirmed on ADC mapping, were virtually absent in the images from the non-prion cases being seen in only one case, a patient with cerebral lymphoma (figure 3) Two other patients, one with LGI1 antibody encephalopathy who had abnormal signal in the swollen left basal ganglion on DWI (figure 4) and the other had a pulvinar sign (figure 5) but ADC did not confirm restriction in either.

No case of CJD had gadolinium enhancement or haemorrhage but this did occur in one case of leukaemia with cortical infiltration.

#### Cerebrospinal fluid examination

CSF protein levels were normal or moderately elevated <1.0 g/L) in both groups of patients (table 4B). The CSF was always acellular (<2×10^6^ cells/L, usually completely acellular) in the patients with sCJD. Three patients with inflammatory conditions there were more than 4×10^6^ cells per L.

The 14-3-3 and elevated S100B proteins were detected in both groups of patients but significantly more frequently in the patients with sCJD, the 14-3-3 protein being a marginally better discriminant. The RT-QuIC, which was determined in 35% of the patients, was invariably negative in the patients with an alternative diagnosis and positive in 89% of the patients with sCJD.

#### EEG

The first EEG obtained did not discriminate between the two groups with the exception of the presence of periodic complexes which were found in about a third of patients with sCJD (table 4C). However, a similar proportion of the non-CJD cases had sharp components superimposed on slow waves variously reported as epileptic discharges or evolving periodic complexes. In most cases only one EEG was obtained.

## Discussion

Of the neurodegenerative disorders, sCJD is a rare diagnosis. Most general neurologists in the UK will only see a case of sCJD every 1–5 years and without a highly specific test, diagnosis has relied on criteria primarily designed for epidemiology research to distinguish between probable sCJD and other more common neurodegenerative disorders. Prior to 2010, these criteria depended on the rapidity of the cognitive decline, the presence of myoclonus, ataxia, alteration of the signal in the basal ganglia on MRI using initially FLAIR and diffusion restriction, the detection of 14-3-3 protein in the CSF and that no alternative diagnosis has been found. More recently, the MRI criteria have been extended to abnormality of cortical signal on DWI from two or more areas.^3^ These criteria have been used throughout the period over which the patients in the present report were studied. However, after the completion of this study, the RT-QuIC, using a variety of methods, has been added to the criteria and its presence is considered sufficient to make a diagnosis of probable sCJD in any neurodegenerative syndrome;^10^ this parameter has been obtained retrospectively in a proportion of the patients in whom a CSF sample obtained in the appropriate container existed after the test became clinically available in the present study. Our study was not designed or statistically powered to consider diagnostic test performance in pathological and Western blot defined subgroups of CJD.

This paper shows that neurologists were successful in accurately diagnosing sCJD in most cases in this series. Nevertheless, the delay in diagnosis was substantial the median time being over 3 months in a disease where 50% of patients die within 5 months (table 1). This probably reflects multiple effects, including non-specific initial symptoms, the reluctance of clinicians to make a diagnosis for which there is no treatment, misinterpretation of the MRI and the delay in obtaining certain results particularly immunological tests. In the UK, most patients who have sCJD are screened for paraneoplastic antibodies and many have whole body FDG PET seeking occult neoplasia. In this series, there were five patients with such antibodies; in the three with LGI1 antibodies, all were diagnosed after referral on the clinical picture with faciobrachial seizures, a clinical picture that never occurred in patients with sCJD. The other two patients (one with PERM due to GLY1R antibodies, the other with NMDA antibodies) did have features likely to be confused with sCJD. Genetic studies that included a search for the commoner mutations causing cognitive decline revealed three patients with mutations; there were pointers to the correct diagnosis in all three (*C9orf72* and *CSF1R*) on neurophysiology testing and MRI.

Distinguishing sCJD from DLB and AD can be difficult, particularly in those patients with rapid cognitive decline. It is thus not surprising that these two diagnoses were the most frequent mimics of sCJD in the present series. Focal atrophy of the hippocampi and/or parietal region on MRI and dopamine reuptake scanning was of some help in separating patients with AD, including those with a more posterior cortical picture, and DLB from those with sCJD. More recently, CSF measurement of the tau/amyloid β protein ratio has been shown to have a high sensitivity for diagnosis of AD but was not assessed in most of the cases in this series.^15^ Similarly, amyloid beta ligand PET was not in routine clinical use during the course of this study.

It is clear that the MRI of the brain is the most useful test with which to screen any patient who has a clinical picture that could be due to sCJD. MRI is usually rapidly available in hospitals and a DWI sequence can be obtained even in a disturbed patient because acquisition times are short. Restricted diffusion confirmed on ADC mapping in the present series had a sensitivity of 93% and a specificity of nearly 100% (one case had of lymphoma had partial confirmation of restriction). Unfortunately, the classical features of sCJD with restricted diffusion in the cortex, basal ganglia and thalamus are often not detected by the radiologist outside specialist centres, thus delaying a diagnosis and a fruitless search for an alternative.^5^ Conversely, if basal ganglia or cortical signal abnormality is detected but associated with parenchymal swelling, alternative diagnoses such as encephalitis or lymphoma should be considered: parenchymal swelling is not a feature of sCJD MRI. We show that over-reliance on the detection of the 14-3-3 proteins in the CSF can result in an incorrect diagnosis of sCJD as this is a non-specific marker of neurodegeneration.

The recent introduction of the various forms of RT-QuIC into clinical practice promises to make the clinical diagnosis of sCJD more secure.^16–18^ In this series, this test was never positive in the cases that were proven to have an alternative diagnosis and had an extremely high sensitivity for detection of sCJD (87%). These results are consistent with published series revealing a sensitivity of between 82% and 96% and very high specificity presumably reflecting the different forms of recombinant protein used and biological factors of the patients such as distribution of codon 129 genotype.^9 18 19^ Finally, in this paper, no patient with definite CJD had a combination of a negative RT-QuIC and negative DWI sequences on MRI.

## Conclusion

In practical terms, we suggest that the MRI using DWI sequences should be obtained in all patients who have a story of a rapidly progressive neurological syndrome. Restricted diffusion in the basal ganglia and/or more than one cortical region, absence of gadolinium enhancement, with sparing of the white matter points strongly to a diagnosis of sCJD in a patient with an appropriate clinical picture. The RT-QuIC should be obtained on the CSF in all those with features compatible with sCJD and especially those with a scan suggestive of that diagnosis. While overall diagnostic accuracy now appears to be excellent, we still make early diagnoses in a small proportion, largely because of delays in the initial suspicion of CJD. Hopefully direct detection of abnormal prions in peripheral biofluids like blood or urine will ultimately simplify the diagnosis of sCJD a situation that may have been achieved with vCJD.^20–22^
